# Is the Application of Artificial Intelligence-Based Competency Assessments for Selecting Clinical Nurses Acceptable? A Qualitative Study

**DOI:** 10.1097/jnr.0000000000000768

**Published:** 2026-08-12

**Authors:** Hyeongsuk LEE, Hyeongju RYU, Hye Jin YOO

**Affiliations:** 1 College of Nursing, Research Institute of AI and Nursing Science, Gachon University, Incheon, Republic of Korea; 2 Department of Nursing, and Research Institute for Biomedical Health Science, Konkuk University, Chungju, Republic of Korea; 3 College of Nursing Science, Kyung Hee University, Seoul, Republic of Korea; 4 Graduate School of Nursing, Kyung Hee University, Seoul, Republic of Korea

**Keywords:** artificial intelligence, clinical competence, nurses, qualitative research

## Abstract

**Background:**

Artificial intelligence (AI)-based competency assessments have been incorporated into nurse recruitment processes in South Korea; however, nursing applicants' and educators’ experiences with these assessments, as well as their perceptions of the effectiveness of these assessments in nurse selection, have not been examined.

**Purpose:**

The aim of this study was to comprehensively explore the perceptions of nursing applicants and educators of the effectiveness of AI-based competency assessments in the process of selecting new nurses for patient care roles.

**Methods:**

A qualitative descriptive design was used in this study. Data were collected between February and August 2024 in South Korea. Semistructured focus group interviews were conducted with 10 nursing applicants and 10 nurse educators, including nursing professors and nurse managers, regarding their experience participating in AI-based competency assessments. Focus group interviews were conducted in five groups, each consisting of three to five participants. Data were analyzed using conventional content analysis. The COREQ (Consolidated Criteria for Reporting Qualitative Research) guidelines were used to assess study rigor.

**Results:**

The analysis of the interviews revealed eight subthemes and four themes derived from 32 codes. The following four themes were identified: (a) doubts about the evaluation method and criteria, (b) efficiency of AI-based competency assessments, (c) challenges in preparing for AI-based competency assessments, and (d) improvements and alternative approaches to nurse selection.

**Conclusions/Implications for practice:**

This study highlights the potential of AI-based competency assessments to improve fairness and efficiency in the process of selecting and hiring new nurses. To ensure validity, developing tailored algorithms that reflect core nursing competencies and establishing clear evaluation criteria is necessary. Also, interdisciplinary collaboration is needed to support the ethical and practical integration of AI in the process of selecting and hiring new nurses.

## Introduction

Medical institutions must secure sufficient numbers of qualified nursing personnel to ensure both patient safety and stable health care system operations (Dall’Ora et al., 2022). This need for adequate nurses is particularly high in South Korea, which has three times more hospital beds than the average for member states of the Organization for Economic Co-operation and Development (OECD, 2021). As part of the nurse recruitment process in South Korea, a national nurse licensing exam for prospective nursing university graduates is conducted annually, and many medical institutions conduct formal, large-scale recruitment and nurse selection procedures (Lee & Choi, 2023).

The rapid advancement of artificial intelligence (AI) technologies has opened new opportunities in human resources management. AI-based competency assessments (AICAs) are a new method that employs AI to vet applicants and select new employees (Kim et al., 2025). AICAs and similar AI-based selection tools have been used in the United States and Japan since 2018, and are increasingly being adopted by institutions in South Korea (Kim & Kwon, 2022). Although the specific content of AICAs varies by developer and target user (e.g., medical institutions), these assessments (as specified by a leading developer in South Korea; Midas IT, 2023) generally consist of a self-introduction, simple video interview, personality test, situational questions, an online AI game (to evaluate reasoning and multitasking), and in-depth questions.

From the perspective of management, the primary goal of using AICAs is to improve corporate productivity by using an automated system to objectively identify applicants who are best qualified to improve human resource utilization and work performance (Black & van Esch, 2020). From the perspective of job applicants, AICAs are new and convenient systems as well as indicators of employer innovativeness; thus, these assessments encourage applicant participation (Horodyski, 2023; Kim et al., 2025). Despite these general perspectives, research concerning the use of AI in recruitment processes presents several problems. First, some applicants may perceive the use of AI in the application process as unfair, which may discourage their participation due to anxiety associated with the use of digital devices (Hockley et al., 2024). Although this discomfort may be present among nursing applicants and educators, the issue has not been examined in the nursing context. Second, in nursing practice, several human interaction-related competencies, e.g., therapeutic communication skills, are recognized as crucial to ensuring quality care and patient safety. However, despite the use of AICAs in nurse selection in South Korea for 4 years, the effectiveness of AICAs in vetting and selecting nurses based on these competencies in the South Korean context has yet to be examined in the literature. Third, although the findings of previous studies (Black & van Esch, 2020; Horodyski, 2023) highlight the advantages of AICA systems, these studies have focused primarily on the perspective of hiring managers.

Therefore, this study was designed to explore the experiences of nursing applicants and nurse educators regarding the use of AICAs in selecting nurses responsible for patient care. The research question applied in this study was “What are the experiences of nursing applicants and educators regarding the use of AICAs during the process of selecting and hiring new nurses?”

## Methods

### Study Design

A qualitative descriptive approach using focus group interviews was employed to explore the experiences of participants with a nurse selection process involving AICA. Study data were analyzed using content analysis.

### Setting and Participants

This study was conducted in South Korea, where tertiary hospitals have recently begun implementing AICAs as part of their selection process for new nursing applicants. New nursing applicants with prior AICA experience, nurse educators (associate and assistant professors) with experience providing AICA-related employment guidance, and nurse managers (e.g., head nurses and nurse team leaders responsible for personnel management) with experience conducting nurse selection processes involving AICAs were recruited as participants. In addition, all of the participants were required, after receiving a detailed explanation of the study procedures, to demonstrate a clear understanding of the study’s purpose and to volunteer to participate. The exclusion criterion was having a hearing or vision impairment that made effective communication impossible.

Purposive and snowball sampling methods were used in the recruitment process, and recruitment notices were posted on online bulletin boards in South Korea. To minimize the potential bias associated with snowball sampling, the recruitment process was purposively designed to ensure participant heterogeneity in terms of both institutional and regional diversity. In addition, to avoid repetitive recruitment via a single platform, purposive sampling was conducted by posting physical recruitment notices at various hospitals and universities. The research team monitored potential sources of bias throughout the recruitment process. Specifically, for the recruitment of nurse managers, the participants were selected using institutional recommendations as well as recruitment notices posted to online bulletin boards by hospitals that had already implemented AICAs. Furthermore, in-depth interviews were conducted with three participants with extensive experience in nursing education and administration to achieve data saturation on the study phenomenon. This study included a total of 10 nursing applicants and 10 nurse educators (i.e., professors and managers). The retention rate was 100%, with no participants dropping out of the study.

### Data Collection

Data were collected from the 20 participants between February and August 2024. Two of the researchers conducted the five semistructured focus group interviews (two with nursing applicants and three with nurse educators), which included three to five participants each. The interviews began only after these researchers had explained the study purpose and the interview and audio-recording processes and had obtained written consent. The interview guide shown in Table 1 was used to guide the interview process, with the semistructured format allowing the interviewers to adapt follow-up questions based on each participant’s role. For example, nurse managers were asked additional questions related to the institution-level implementation of AICAs, decision-making authority, and the practical challenges faced in managing AI-based selection processes. Researcher 1 conducted the interviews, and researcher 2 took the field notes used in the data analysis. Face-to-face interviews were conducted for 60–90 minutes in a quiet seminar room that reflected the participants’ preferences. All of the interviews were audio-recorded, and data saturation was achieved in the fifth group interview. Data saturation was determined as the point at which no new codes or subthemes emerged, and patterns in perspectives were repeated among the nurse managers. Researcher 1 transcribed each interview on the same day it was conducted.

**Table 1:** Interview Guide

Question Type	Question	Participant Group
Opening	Please introduce yourself.	All participants
Introduction	What experiences have you had while preparing for employment as a new nurse?	Applicants
	What is your experience with coaching/counseling nursing students to help them acquire the competencies required to become nurses after graduation?	Professor
	What is your experience coaching/counseling new nurses to help them acquire the necessary competencies after graduation?	Manager
Transition	What are your overall thoughts on introducing an AI-based competency assessment when recruiting nurses?	All participants
Key	What are your thoughts on AI-based competency assessments compared with traditional interviews?	All participants
	What were some of the challenges you faced during AI-based competency assessments, and how did you overcome them?	Applicants, Professor
	What organizational challenges have you encountered in using AI-based competency assessments for nurse selection?	Manager
	What strategies have helped nursing students prepare for AI-based competency assessments?	All participants
	What were the expected effects of introducing AI-based competency assessments into the nursing recruitment process?	Applicants, Professor
	As AI-based competency assessments are now being used in nurse recruitment, how do you think this will influence nursing practice and human resource management in the future?	Manager
	What training/resources are needed to prepare for an AI-based competency assessment?	All participants
	What role do you play in the institutional implementation of AI-based competency assessments?	Manager
Ending	Is there anything you wish to add?	All participants

*Note.* AI = artificial intelligence.

### Data Analysis

The collected data were analyzed using conventional content analysis (Hsieh & Shannon, 2005). Content analysis, mainly used to analyze interview data by developing categories using an inductive coding approach, was deemed appropriate for exploring the AICA-related experiences of the participants.

With regard to the steps used in this analysis, the researchers first read the text, focusing on words, phrases, and sentences considered significant, and generated codes that were subsequently marked and named. Next, two researchers (a) independently reviewed the transcripts multiple times to understand participant experiences; (b) individually created an initial coding frame using Excel software based on the identified significant words, phrases, and sentences; (c) compared the relevance of and differences among the generated codes and grouped related codes into subthemes; (d) compared the relevance of and differences among the subthemes and classified these under a smaller number of higher-level themes; (e) named the themes based on the relevance of codes, subthemes, and themes; (f) composed a report that comprehensively reflected participant statements; and (g) regularly discussed and reached consensus on the process used to analyze data. After separately analyzing the data, the two researchers disagreed regarding the interpretation of certain nuanced emotional expressions (e.g., distinguishing between “anxiety” and “confusion”) and the category classification of several subthemes. These differences were addressed through iterative discussions with the third researcher, who has extensive experience in qualitative research and subject-matter expertise in both nursing education and AI. The third researcher participated in the overall analytic process and facilitated consensus by reviewing divergent interpretations and ensuring consistency across the coding framework. The final themes were confirmed based on consensus among all three researchers.

### Rigor

Rigor was ensured in this study through adherence to the following four criteria. Credibility: Only individuals capable of articulating the study phenomenon in depth were selected as participants. Despite the small number of nurse managers in the sample, all had extensive experience implementing AICAs at their institutions. Further, interviews were conducted in a comfortable environment to encourage participants to express their opinions openly. Fittingness: Data were presented in tables detailing the general characteristics of the participants and collected until saturation (i.e., when no new information emerged). Auditability: The research process was described in detail and adhered to the COREQ checklist (Tong et al., 2007). In addition, all interview data were presented in direct quotation format. Confirmability: Discussions and agreements among researchers were achieved through regular meetings among the researchers to ensure personal judgment and bias did not affect the interviews or the interpretation of results. Furthermore, the participants confirmed the findings reported by the researchers during the member-check process, which involved providing one or two participants from each focus group with a summary of the identified themes and subthemes via email. Their feedback was used to refine the clarity of thematic labels and confirm that the interpretation of results adequately represented their perspectives. Based on their suggestions, minor wording adjustments were made to enhance the accuracy and resonance of the themes.

### Ethical Considerations

Institutional review board approval was obtained before conducting the study (IRB no. 2023-11-029). Before the interviews, the research and recording procedures were explained to the participants, and their written informed consent to participate was obtained. To protect privacy, all of the collected data were anonymized and coded. After completing the study, the participants received gift cards as compensation.

## Results

The average age was 23.8 (*SD*=0.9, range=23–26) years for the 10 nursing applicants and 43.6 (*SD*=7.6, range=33–56) years for the 10 nurse educators (Table 2). Seven (70%) of the nursing applicants and nine (90%) of the nurse educators were female.

**Table 2:** Participant Characteristics (*N*=20)

Characteristic
	Nursing Applicants (*n*=10)	Nurse Educators (*n*=10)
	*n* (%)	*n* (%)
Age (years; *M* and *SD*)	23.8±0.9	43.6±7.6
Range (min-max)	23–26	33–56
Gender		
Male	3 (30.0)	1 (10.0)
Female	7 (70.0)	9 (90.0)
Educational level		
≥ Graduate school	0 (0.0)	10 (100.0)
≤ University	10 (100.0)	0 (0.0)
Position		
Nursing professor	—	7 (70.0)
Nurse manager	—	3 (30.0)
Education and clinical experience of nurse educators (years; *M* and *SD*)	—	18.3±10.5

Eight subthemes associated with four themes were derived from the 32 codes identified in the interview analysis. The four themes, namely “doubts about the evaluation method and criteria,” “efficiency of AI-based competency assessments,” “challenges in preparing for AI-based competency assessments,” and “improvements and alternative approaches to nurse selection” (Table 3), were synthesized from the eight subthemes through continuous discussions among the researchers to capture higher-level conceptual categories. For example, expressions of uncertainty and skepticism regarding the evaluation method used in the AICA were integrated under the “-doubts about the evaluation method and criteria” theme. Next, the relationships between the themes were mapped conceptually to reflect the shared and divergent experiences of the participants. Differences in attitudes between groups, particularly those between nursing applicants and nurse educators, were analyzed to uncover the underlying contextual factors shaping their perceptions regarding the AICA.

**Table 3:** Experience in Introducing AI-Based Competency Assessments for Nurse Selection

Theme	Subtheme	Code
1. Doubts about the evaluation method and criteria	Incompatibility of automated interview approaches with person-centered nursing	An evaluation method that is far from the concept of person-centered nursing (applicant, professor)
		A mechanical method that fails to properly evaluate the human aspect (manager, professor)
		Insufficient reflection of the hospital’s vision (manager, professor)
		Questions that do not match the characteristics of nursing work (professor)
	Confusion compounded by opaque evaluation criteria	Lack of understanding of the introduction of AI interviews (applicant, professor)
		Rumors about the evaluation criteria (applicant)
		Specific undisclosed details about acceptance or rejection (applicant)
		Nursing students struggling to prepare for employment (professor)
2. Efficiency of AI-based competency assessments	Opportunities to flexibly participate in job interviews and self-assess one’s competencies	A method that does not require long-distance travel (applicant, professor)
		Efficiency in selecting and utilizing interview time (manager)
		Allows preparation for employment at various hospitals (applicant)
		Provides time to think deeply about oneself (applicant)
	As a screening tool to select excellent nursing personnel	A valuable filtering tool for large-scale recruitment (Manager)
		Multifaceted evaluation for aspects including concentration, agility, and personality (manager, professor)
		Easily assessing applicants’ capabilities before a face-to-face interview (manager)
		Securing nursing time by reducing the time spent on interview evaluation (manager)
3. Challenges in preparing for AI-based competency assessments	Anxiety about the influence of external environmental factors	Variables such as unstable network, lighting, and noise (applicant)
		Mental burden of having to consider many variables (applicant)
		Evaluation results vary depending on the educational environment related to the use of information technology (manager, professor)
		Concerns about evaluation being affected by information support gaps (manager, professor)
	A dehumanizing reality—practicing for a machine with no interpersonal connection	Focus on skills-based preparation while omitting the core of nursing, such as humanity and interaction (professor)
		Speaking practice tailored to AI (applicant)
		Preparation focused on skills such as pupils and mouth shape (applicant)
4. Improvements and alternative approaches to nurse selection	Development and ongoing management of an AI program tailored for selecting qualified nursing personnel	Proposal for development of an AI program focused on nursing (applicant, manager, professor)
		Need to develop programs based on interaction, such as communication and empathy (applicant, professor)
		Using AI-based competency assessments to assist in face-to-face interviews (manager, professor)
		Building sophisticated algorithms based on big data (manager)
		Continuous feedback on the appropriateness of the evaluation method (manager, professor)
	Efforts to increase adaptation to the new selection system	Sharing information on clear AI-based competency assessment guidelines (applicant, professor)
		Building a network of seniors and juniors to prepare for employment (Professor)
		Building an education system using AI programs (applicant, professor)
		Active efforts to apply nursing education to enhance digital literacy competencies (professor)

*Note.* AI = artificial intelligence.

### Theme 1: Doubts About the Evaluation Method and Criteria

This theme reflects uncertainty and skepticism regarding AICA fairness and appropriateness, particularly in terms of alignment with nursing values and the clarity of evaluation standards. Since the COVID-19 pandemic, many medical institutions have introduced AICAs to evaluate individual competencies when hiring nurses. However, the lack of clarity regarding the evaluation methodology and criteria used by AICA systems has created confusion for applicants as well as nurse educators.

#### Incompatibility of Automated Interview Approaches With Person-Centered Nursing

The nursing applicants considered AICA to be incongruous with their learned concept of person-centered care. After interfacing with an AICA program via a computer for nearly 1 hour during a previous job recruitment process, they felt this method was not suitable for nursing, which prioritizes interpersonal interactions.

*I learned that nursing is a human-to-human interaction and a noble task. It is a contradiction to use AI to select nurses, who care for patients.* (Nursing applicant group 1-1)

The nursing professors questioned why competencies essential to patient care, such as nursing and counseling skills, were insufficiently represented in the AICA. The nurse managers emphasized that their hospital’s mission was inadequately represented in the AICA and raised concerns about potential discord between organizational values and evaluation criteria.

*I think AI-based evaluations have a strong mechanical aspect that makes it difficult to reveal the warm human side of the students.* (Professor group 1-2)

*Unfortunately, the mission pursued by each hospital is not reflected in the evaluation criteria. I think many questions do not fit the characteristics of nursing.* (Manager group 1-2)

#### Confusion Compounded by Opaque Evaluation Criteria

The nursing applicants expressed uncertainties regarding the evaluation criteria used in AICAs. They were frustrated because they did not know the exact assessment criteria used, the bases of the assessment outcomes, or whether the evaluation was made by a machine or a person.

*There are many rumors regarding the evaluation criteria. This was frustrating because both those who passed and those who failed did not know the reason*. (Nursing applicant group 2-4)

Nurse educators also expressed uncertainty about the evaluation criteria used. They stated that, after the introduction of the AICA, there was a need to evaluate its effect in terms of nurse adaptation and resignations to determine its validity as a nurse selection tool.

*I think we need to establish an evaluation standard and have AI operate based on that standard. But I think the most difficult thing right now is that the standard itself is unclear*. (Professor group 2-3)

*AI-based assessments have only been introduced for a few years; therefore, their impact on the correlation between nurse turnover and adaptation has not yet been analyzed. I expect AI-based evaluations to filter out many applicants rather than provide a high-quality evaluation of nursing competency*. (Manager group 1-1)

### Theme 2: Efficiency of AI-Based Competency Assessments

This theme reflects recognition of the logistical advantages and potential usefulness of AICA in streamlining the nurse selection process. The nursing applicants acknowledged the convenience of the AICA due to the lack of time and space restrictions. The nurse educators noted that the use of the AICA allows for more applicants in the recruitment process while simultaneously reducing the burden on human resource staff to assess each applicant.

#### Opportunities to Flexibly Participate in Job Interviews and Self-assess One’s Competencies

Those nursing applicants scheduled for multiple job interviews were able to complete each at their convenience at any location with an internet connection.

*When I was in my 4th year, if my classes and (nurse) job interviews overlapped, I had to give one of them up. However, I like that the AICA allows me to participate conveniently at any time and place I want, thus avoiding skipping class*. (Nursing applicant group 1-3)

The nursing professors stated that the AICA lets students faithfully attend their final semester classes and prepare for employment simultaneously.

*I like that students can choose to participate in job interviews at any time convenient for them, with no interruption to their class schedule*. (Professor group 2-1)

Moreover, in spite of the difficulties presented by the new evaluation methods, the nursing applicants learned more about themselves through the analysis and practice components of the AICA.

*I never thought deeply about myself during this time. I tried my best to adapt to the AI-based system and worked hard to determine whether I was the right person to become a nurse. Although difficult, it ultimately became an opportunity to discover who I was*. (Nursing applicant group 1-2)

The nurse managers acknowledged the AICA as a useful tool for elucidating applicant attitudes.

*I think it is effective in checking the consistency of applicant attitudes*. (Manager group 1-1)

#### As a Screening Tool to Select Excellent Nursing Personnel

All of the participants recognized AICA as a useful tool for expanding the pool of excellent talent by significantly expanding the number of applicants. The nurse educators stated the ability to perform primary screening with AI as a strength in terms of improving applicant vetting efficiency.

*The fact that it (AICA) gave many applicants a chance to participate is attractive*. (Nursing applicant group 2-4)

*Using an AI program, multifaceted evaluations such as concentration, agility, and personality are possible, and successful applicants can be filtered based on analysis algorithms*. (Professor group 2-2)

Using AI for competency evaluation, the nurse managers stated that they were able to reduce personnel-related costs and work fatigue by reducing the number of personnel involved in job interview evaluations.

*I can prepare for face-to-face interviews by understanding applicant capabilities in advance. Thanks to AI, the burden of human resource evaluation work that I am responsible for has been reduced, and the fatigue caused by this has been alleviated. I think an environment has been created where I can focus more on my main job, nursing*. (Manager group 1-2)

### Theme 3: Challenges in Preparing for AI-Based Competency Assessments

This theme describes the practical and emotional difficulties participants faced while preparing for AICAs, including technical barriers and a lack of human interaction. The nursing applicants reported that, while preparing for their AICAs, they experienced difficulties due to factors unrelated to personal competency, such as internet connectivity issues, information gaps, and nurse educators’ lack of guidance and experience. Applicants and nurse educators both invested considerable thought and effort into finding ways to pass the AICA.

#### Anxiety About the Influence of External Environmental Factors

Applicants were concerned about environmental factors such as network stability, lighting, and noise, with a stable network necessary to ensure the connection was not lost during the evaluation, and proper lighting and noise blocking necessary to minimize distractions.

*Because so many people were connected at once, it kept crashing for three hours. I logged in early in the morning when there were not too many people, and I searched for a well-lit and soundproof internet café to avoid technical issues. I paid attention to all of the variables that could have an impact*. (Nursing applicant group 1-5)

Nurse educators expressed concerns that applicants’ results could be influenced by the educational environment in terms of factors such as the ability of applicants to participate in mock evaluations using AI.

*There is bound to be a difference between students who can freely use information technology and those who cannot. I am concerned that an imbalance in information support may lead to fairness issues for applicants*. (Professor group 2-3, Manager group 1-1)

#### A Dehumanizing Reality—Practicing for a Machine With No Interpersonal Connection

AICAs are based on one-sided communications with a computer without interpersonal interaction. Consequently, nursing applicants reported that they relied on hearsay and focused on practicing speaking based on erroneous evaluation criteria.

*I heard that because the evaluation is done by a robot, you have to be as consistent as possible to obtain a high score. Instead of speaking in my own style, I practiced speaking in a stereotypical way, like a robot: “Hello, this is XXX.”* (Nursing applicant group 1-1)

Nurse educators highlighted the omission of foundational nursing aspects, such as humans and interactions, during the nurse selection process. They considered the purpose of the AICA and hoped applicants would prepare based on their thoughts and experiences regarding patient care.

*I was saddened to see the students focus on AI tools, personal appearance, and skills in using AI*. (Professor group 2-1)

*It is important to explore the essence of nursing and express one’s thoughts clearly. However, applicants tend to focus on improving their skills only*. (Manager group 1-2)

### Theme 4: Improvements and Alternative Approaches to Nurse Selection

This theme addresses the suggestions of the participants for enhancing AICA in terms of developing nursing-specific AI systems and helping applicants adapt to the new selection method. They expressed hope that the AICA would facilitate face-to-face interviews when clear information regarding the evaluation guidelines used at each medical institution is available.

#### Development and Ongoing Management of an AI Program Tailored for Selecting Qualified Nursing Personnel

Both nursing applicants and nurse educators shared that the current AICA program was not sufficiently specialized for new nurse selection. They agreed AI programs should be developed to consider communication, empathy, and other interactive aspects central to nursing competencies.

*I do not know how the therapeutic communication, empathy, and consideration aspects of nursing that I have learned are addressed in the AI-based competency assessment. If a nurse-tailored AI program is developed, the validity of AI-based competency assessments in the nurse recruitment process could be enhanced*. (Nursing applicant group 2-4)

The nurse educators emphasized the need to develop data-driven algorithms for nurse selection and to review and update AI-based evaluation methods periodically. They expected the AICA to provide useful data to help evaluators select excellent personnel during face-to-face interviews.

*It is necessary to build an algorithm to select excellent nurses based on applicant big data, and to periodically review and update the appropriateness of the evaluation method*. (Professor group 1-4)

*I believe that the results of applicant evaluations using AI will provide useful basic data for face-to-face interviews*. (Manager group 1-3)

#### Efforts to Increase Adaptation to the New Selection System

Nursing applicants and nursing professors wanted to share official information about the AICA evaluation guidelines and build senior–junior networking. They identified clear standards and thorough preparation as effective methods to motivate applicants to pursue nursing careers.

*I feel sorry for the students who were stressed during the preparation because of vague standards. I plan to connect with seniors who have participated in AI-based programs and successfully secured nursing positions to receive mentoring from them*. (Professor group 1-2)

Both the nursing applicants and nurse managers expressed the need for an educational intervention that incorporates AI program preparation to further foster digital literacy in nursing universities.

*I think that if I am exposed to AI programs in university, I will not be embarrassed and will adapt to the system well*. (Nursing applicant group 1-3)

*Because information processing skills such as computer use are important for nurses these days, it would be beneficial if nursing education included programs designed to equip nurses with digital literacy*. (Manager group 1-1)

## Discussion

While the integration of AICAs into the nurse selection process has begun to transform nurse recruitment, their implications for health care practice remain underexplored. In this study, the perspectives of nursing applicants and educators were explored regarding the use of AICA in nursing, a topic currently addressed by limited empirical research. This qualitative exploration of the perceptions of nursing applicants and educators identified key themes that reflect the opportunities and challenges of AICA in the nursing context. Specifically, while acknowledging the logistical efficiency that AICA offers in large-scale recruitment processes, both nursing applicants and educators expressed concerns about the ambiguities inherent in evaluation methods and the misalignment of these methods with core nursing values.

The participants perceived AICAs as valuable for improving fairness and efficiency in nurse selection. They highlighted that AICA systems enable the evaluation of concentration, agility, and personality, all of which are difficult to assess in traditional, short face-to-face interviews. The standardized, data-driven nature of AICAs was identified as enhancing selection process objectivity and reducing bias, which aligns with previous findings on AI’s potential to promote fairness and efficiency (Dima et al., 2024; Goswami et al., 2023). Furthermore, the participants identified that the flexibility of AICAs, unrestricted by time or location, increases accessibility for nursing applicants and supports scalability in large-scale recruitment processes. However, several challenges also emerged. First, the participants frequently cited gaps arising from environmental factors during AICA preparation. They reported difficulties related to unstable network connections, poor lighting, and limited access to essential hardware such as cameras and speakers. These external variables undermined the ability of the nursing applicants to prepare for the evaluation. Therefore, developing a robust hardware infrastructure that supports stable access and minimizes environmental variability is essential.

In addition, participants noted that the lack of transparency regarding AICA evaluation criteria was a source of significant confusion and stress during preparations. The nursing applicants and nurse educators pointed out that unclear standards undermined preparation fairness and trust in the selection process. Considering that nursing competency is largely intangible and difficult to measure using fixed indicators, establishing transparent and consistent evaluation criteria is essential to ensuring fairness and credibility (Song et al., 2024).

Also, the participants expressed concerns about the ability of the AICA to adequately capture nursing competencies such as clinical decision-making, therapeutic communication, and empathy, all of which are central to professional nursing practice (Alnjadat et al., 2024). Given that nursing is inherently relational and context-dependent, the participants questioned the appropriateness of relying solely on AICAs for selecting and hiring new nurses. They called for integrating assessment elements that better reflect human interaction and caregiving-related behaviors. These concerns reflect broader ethical issues related to AI-based selection. Scholars warn that overreliance on automation may erode human dignity and judgment, particularly in empathy-driven fields (e.g., nursing) where AI’s lack of emotional and contextual understanding poses particularly significant risks (Amin et al., 2025; Hockley et al., 2024; Hunkenschroer & Kriebitz, 2023). Therefore, AI should be positioned as a supportive tool that complements rather than replaces human evaluators in the selection process.

The findings of this study point to the need to regularly refine nursing-related AICA algorithms to reflect the multifaceted competencies required in nursing practice more accurately. The nurse educators in this study emphasized that algorithm development should leverage the big data accumulated from nursing applicants to capture qualities that define professional nursing practice, such as cognitive ability, interpersonal skills, communication, and ethical reasoning (Yakusheva et al., 2025). Therefore, collaboration between AI developers and medical institutions will be essential to address these dimensions and design contextually relevant systems.

Furthermore, the nurse educators in this study were cautious about positioning AICAs as standalone tools. Rather, they recommended using AICA results to inform and enrich traditional selection methods such as face-to-face interviews. For example, AICA outputs were considered helpful in formulating interview questions targeting specific behavioral or cognitive areas flagged by the system. This aligns with existing arguments that AI should serve, particularly in person-centered professions such as nursing, as a supportive rather than substitutive tool in human resource decision-making (Hunkenschroer & Kriebitz, 2023). Institutional readiness, including a clear understanding of AICA operations and how AICA-generated data are interpreted, remains crucial for effective implementation (Goswami et al., 2023).

Taken together, these insights reflect the importance of aligning technological innovation with professional values. The effectiveness of AICAs will depend on their technical sophistication, integration with human judgment and ethical oversight throughout the selection process (Shahzad et al., 2023). Taken as a whole, the findings support the continued development of contextually relevant, ethical AI tools to enhance the process of selecting and hiring new nurses.

### Limitations

Despite its contributions to clarifying the effectiveness of AICAs, this study was limited by the narrow cultural and institutional settings considered, which may restrict the generalizability of the findings to other health care contexts. Also, the lack of diversity in the AICA experience within the sample (all of the participants had recent experience with AICA systems) potentially narrowed the scope of perspectives. In addition, the qualitative interview approach used cannot provide an objective assessment of AICA's effectiveness. Finally, given the rapid evolution of AI technologies, the findings of this study may be time-sensitive. Future research into this topic should incorporate diverse user groups and longitudinal or mixed-method approaches to enhance robustness and applicability.

### Implication for Practice

To ensure that nursing-specific competencies are adequately captured and given appropriate consideration, AI-based selection systems should be developed in a collaborative process that includes experts from the fields of nursing, science, and information technology. Moreover, for AICA to function as a strategic tool rather than just a screening method, a dynamic feedback mechanism responsive to changes in the health care environment should be implemented, allowing medical institutions and universities to provide standardized AICA preparation guidelines and evaluation criteria to be regularly refined to ensure fairness and transparency. Finally, to reduce disparities in digital readiness, nursing education should include mock assessments and AI-related content to foster digital literacy.

### Conclusions

The potential benefits and limitations of employing AICAs in the nurse selection process are highlighted in this study. While the participants acknowledged the advantages of AICA in terms of enhancing efficiency and objectivity, critical concerns were raised regarding its ability to reflect essential nursing competencies and ensure equitable access. To support effective AICA adoption, health care institutions should collaborate with nursing and AI experts to develop nurse-specific competency models and algorithms. Nursing schools should consider incorporating AICA preparation guidance into their curricula to strengthen students’ digital literacy and familiarity with AI-based systems. Furthermore, policymakers and hospital administrators should work to establish standardized guidelines and robust technical infrastructures to promote fairness and reduce environmental disparities in AI-based assessments. The findings demonstrate the importance of aligning AI-driven selection tools with the relational and context-sensitive nature of nursing practice, as well as offer foundational insights for making continued improvements in the future.
